# Allergic diseases of the skin and drug allergies – 2029. Prevalence and factors of food allergy among school-age children in Southern China

**DOI:** 10.1186/1939-4551-6-S1-P115

**Published:** 2013-04-23

**Authors:** Mo Xian, Jing Li

**Affiliations:** 1 Department of Allergy and Clinical Immunology, Guangzhou Institute of Respiratory Disease, China; 2 Department of Allergy and Clinical Immunology, State Key Laboratory of Respiratory Disease, the First Affiliated Hospital of Guangzhou Medical College, Guangzhou, China

## Background

Food allergies (FA) is influenced by genetic and environmental factors. The aim of the present study was to investigate the prevalence and factors of FA among school-age children in southern China.

## Methods

5836 children aged 7–12 years living in Guang Zhou, China participated in this study. A cross-sectional parent questionnaire survey was conducted. Families with 189 children confirmed as FA (according to self report and doctor’s diagnosis) and other 213 randomly selected health children completed a standardized questionnaire related to respiratory and food allergic symptoms, family history of allergic diseases, smoking history, environmental exposure, and eating behaviors. They underwent skin-prick tests (SPTs) with 24 common food allergens and aeroallergens. Blood samples were collected from 186 children with FA and 207 health child for peripheral blood eosinophils (EOS) analysis and specific IgE (sIgE) measurements against 27 common food allergens and 6 aeroallergens.

## Results

A total of 5542 out of 5836 questionnaires (94.96%) were returned. 3.41% children were reported to have FA and were also ever diagnosed as FA by doctor. The five leading allergic foods reported were shrimp(24.26%), crab (14.85%), cow’s milk (8.91%), chicken eggs (7.42%) and fish (5.29%). The most commonly reported symptom was a skin rash (63.28%), followed by upper respiratory symptoms (37.5%). There was no significant difference between children with FA and health children on EOS level (*p*>0.05). EitherSPTs or sIgE analysis showed that the main allergic foods were shrimp (*p*<0.05) and crab (*p*<0.05). Earlier age (<8 years), family history of allergic diseases, frequently migrating during infancy, often exposed to smoke environment, frequently eating beef suet and parents with higher education were associated with increased risk of SPT and sIgE positivity. However, earlier going to nursery (2 years old) may decreased the risk of SPT positivity.

## Conclusions

The prevalence of FA confirmed on self-report and doctor’s diagnosis was 3.41% in southern China. The most common causative food were shrimp and crab. Earlier age (<8 years), family history of allergic diseases, frequently migrating during infancy, often exposed to smoke environment, frequently eating beef suet and parents with higher education are risk factors for food allergen sensitizations, whereas earlier going to nursery (2 years old) may be the protective factor.

